# Association of Polymorphisms in the Atrial Natriuretic Factor Gene with the Risk of Essential Hypertension: A Systematic Review and Meta-Analysis

**DOI:** 10.3390/ijerph13050458

**Published:** 2016-04-29

**Authors:** Jinyao Wang, Zhenkun Wang, Chuanhua Yu

**Affiliations:** 1 Department of Epidemiology and Biostatistics, School of Public Health, Wuhan University, 115 Donghu Road, Wuhan 430071, China; jinjinyao456@163.com (J.W.); wongzhenkun@gmail.com (Z.W.); 2 Global Health Institute, Wuhan University, 115 Donghu Road, Wuhan 430071, China

**Keywords:** ANP, polymorphism, essential hypertension, meta-analysis

## Abstract

*Background*: Studies evaluating the association between the atrial natriuretic peptide (ANP) genetic polymorphism and the risk of essential hypertension (EH) have reported inconsistent results. The aim of this meta-analysis was to provide a more reliable estimation of the possible relationship between the atrial natriuretic peptide genetic polymorphism and the risk of essential hypertension (EH). *Methods*: Relevant articles were searched to identify all case-control or cohort design studies of the associations between ANP polymorphism and EH. The heterogeneity was checked using the Q test and the inconsistent index (*I*^2^). The odds ratio (OR) test and 95% confidence interval (CI) were calculated in a fixed or random effects model to evaluate the strength of association. Begg’s test and Egger’s test were applied to evaluate the publication bias. *Results*: A total of 25 case-control studies including 5520 cases and 5210 controls exploring the association between ANP polymorphism and EH were available for this meta-analysis. No significant association between the T2238C polymorphism and overall EH risk under the five genetic models was found (C *vs.* T: OR = 1.1, 95%CI = 0.94–1.2, *p* = 0.38; TC *vs.* TT: OR = 1.1, 95%CI = 0.88–1.5, *p* = 0.32; CC *vs.* TT: OR = 1.3, 95%CI = 0.90–1.9, *p* = 0.16; (CC + TC) *vs.* TT: OR = 1.1, 95%CI = 0.88–1.4, *p* = 0.35; CC *vs.* (TT + TC): OR = 1.1, 95%CI = 0.83–1.4, *p* = 0.55). We also found that the G1837A polymorphism had no significant association with overall EH risk (A *vs.* G: OR = 1.3, 95%CI = 0.96–1.9, *p* = 0.090; GA *vs.* GG: OR = 1.5, 95%CI = 0.83–2.6, *p* = 0.19; AA *vs.* GG: OR = 0.87, 95%CI = 0.34–2.3, *p* = 0.78; (AA + GA) *vs.* GG: OR = 1.5, 95%CI = 0.86–2.5, *p* = 0.17; AA *vs.* (GG + GA): OR = 1.3, 95%CI = 0.85–2.0, *p* = 0.22). In the analysis of the T1766C polymorphism, after removing the study of Nkeh, the 1766C allele suggested a protective effect in the model of TC *vs.* TT (OR = 0.64, 95%CI = 0.47–0.86, *p* = 0.003) and (CC + TC) *vs.* TT (OR = 0.64, 95%CI = 0.48–0.87, *p* = 0.004). *Conclusions*: This meta-analysis suggested that no significant relationships between ANP T2238C, G1837A gene polymorphisms and the risk of essential hypertension exist. Conversely, the ANP T1766C gene polymorphism may be associated with the risk of essential hypertension, and the 1766C allele may be a protective factor against EH. However, due to the number of limited articles on the T1766C polymorphisms, further studies are still needed to accurately prove the association between the T1766C gene polymorphism and the risk of essential hypertension.

## 1. Introduction

Cardiovascular disease (CVD), including essential hypertension (EH), is the leading cause of mortality throughout the world [1]. Among the important worldwide public-health challenges, hypertension has become an independent predisposing factor for many cardiovascular diseases, including coronary heart disease, heart failure stroke and many other serious cardiovascular diseases. It is estimated that hypertension is the third most important risk factor for disability-adjusted life-years [2,3]. Although mortality caused by cardiovascular disease has recently declined, the burden of CVD remains high [4]. Hypertension is a complex disease regulated by many interactional systems that have remained unclear until now [5]. Hypertension is likely to be a type of multifactorial, polygenic and genetic disorder influenced by genetic variations [5,6], and there are some reports on possible candidate genes [7]. Genetic elements played a vital role in the range of blood pressure [8] in human essential hypertension; blood ANP levels are considered to be higher than those in normal subjects [9]. In recent years, many studies have discussed the relationships between genetic polymorphisms and essential hypertension, but some of the conclusions are inconsistent and unconvincing.

Atrial natriuretic peptide (ANP), which is also called atrial natriuretic factor (ANF), is a cardiac hormone that is synthesized and secreted in cardiac atrial [9,10,11]. The main physiological role of ANP is to make vascular smooth muscle diastolic and induce apoptosis in cultured cardiac myocytes; in addition, ANP can inhibit rennin-angiotensin-aldosterone and myocardial contractile activity [12,13]. ANP plays an important role in the regulation of blood pressure [14]. In order to provide evidence for the prevention of essential hypertension, many researchers have conducted a series of studies exploring the potential relationships between atrial natriuretic peptide (ANP) genetic polymorphism and essential hypertension [7,15,16]. According to existing studies, several candidate genes have been identified as risk factors of EH; the human ANP gene may be a possible candidate gene contributing to the risk of EH or other cardiovascular diseases [17,18]. Consequently, the current meta-analysis was conducted to examine whether the ANP polymorphisms are associated with patients with essential hypertension.

## 2. Materials and Methods

### 2.1. Literature Search Strategy

We systematically searched PubMed, the Cochrane Library, Wiley, Embase, China National Knowledge Infrastructure (CNKI), and the Chinese WanFang Database for reports of populations based on case-control or cohort design studies published before 1 December 2015. The databases were searched by two authors independently using the following keywords: (“essential hypertension” or “primary hypertension” or “hypertension” or “blood pressure” or “arterial pressure”) AND (“atrial natriuretic factor” or ‘‘ANF’’ or ‘‘atrial natriuretic peptide’’ or ‘‘ANP’’ or ‘‘atrial natriuretic hormone’’ or “ANH” or ‘‘natriuretic peptides’’ or “NPPA” or“ natriuretic peptide precursor A”) AND (‘‘mutation’’ or ‘‘polymorphism, genetic’’ or ‘‘variation’’ or ‘‘polymorphism’’ ‘‘polymorphism, single nucleotide” or ‘‘single nucleotide polymorphism’’ or ‘‘SNP’’ or ‘‘variant’’ or “alleles” or “allele” or “genotype”). We also performed a manual search of the reference lists from relevant articles to find other potential articles. The search was conducted on studies published in English and Chinese.

### 2.2. Inclusion Criteria

Studies that met the following criteria were included:

(1) studies of case-control or cohort design studies;
(2) studies investigating the association between ANP polymorphism and essential hypertension;
(3) full-text articles; and
(4) hypertension was defined as at least three consecutive systolic blood pressure (SBP) measurements ≥ 140 mmHg or diastolic blood pressure (DBP) measurement ≥ 90 mmHg, or receiving antihypertensive pharmacotherapy treatment for at least 1 year; controls were healthy individuals in the same period.

### 2.3. Exclusion Criteria

Studies that did not meet the following criteria were excluded:

(1) duplicated studies;
(2) reviews and literature without detailed genotype data;
(3) studies with no controls;
(4) unpublished articles, abstracts and comments;
(5) subjects in the study were not human; and
(6) SBP < 140 mmHg or DBP < 90 mmHg in cases or secondary hypertension or other serious cardiovascular disease of cases were excluded.

### 2.4. Data Extraction

The following data were independently extracted by two reviewers, and disagreements between the two reviews were resolved through discussion until the reviewers reached a consensus. The data extraction included: the first author’s name, publication year, country, ethnicity, the number of cases and controls the sources of the subjects, genotyping methods, quality score, genotype distribution and allele frequency in cases and controls, and the Hardy-Weinberg equilibrium (HWE, *p* < 0.05 was considered a significant difference from HWE).

### 2.5. Quality Assessment of the Included Studies

The quality of the included studies was independently assessed by two reviewers, and disagreements between the two reviews were resolved through discussion until the reviewers reached a consensus. The quality of the included studies was evaluated using the Newcastle–Ottawa quality assessment scale [19]. The scale includes a total of three categories and eight entries. The number of stars represent the quality of studies. The highest quality research can be granted ten stars. Studies with six stars or higher than six stars were considered high quality.

### 2.6. Statistical Analysis

The STATA 12.0 software (Stata, College Station, TX, USA) was chosen as the statistical analysis software for data management. To evaluate the associations between the ANP T2238C, G1837A and T1766C polymorphisms and the risk of EH, odds ratios (ORs) and 95% confidence intervals (95%CI) were calculated using five models, including an additive model (C *vs.* T), co-dominant model (TC *vs.* TT; CC *vs.* TT), dominant model ((CC + TC) *vs.* TT) and recessive model (CC *vs.* (TT + TC)) of the T2238C polymorphism and T1766C polymorphism. Pooled OR and 95%CI were also calculated under five genetic models including an additive model (A *vs.* G), co-dominant model (GA *vs.* GG; AA *vs.* GG), dominant model ((AA + GA) *vs.* GG) and recessive model (AA *vs.* (GG + GA)) of the G1837A polymorphism. *p* values and *I*^2^ were calculated using the Q-test. The *I*^2^ = [100% × (Q − df/Q)] test for heterogeneity between the results of different studies was conducted. The fixed effects model was used if *p* > 0.10 and *I*^2^ < 50%; the pooled OR and corresponding 95%CI were calculated using the Mantel-Haenszel method. Otherwise, a random effects model using the DerSimonian-Laird method was conducted to evaluate the pooled OR value. Begg’s test and Egger’s test were applied to evaluate the publication bias. *p* < 0.1 indicated that there was significant publication bias, and a relevant funnel plot was drawn.

## 3. Results

### 3.1. Characteristics of the Data Included in the Meta-Analysis

**Figure 1:**
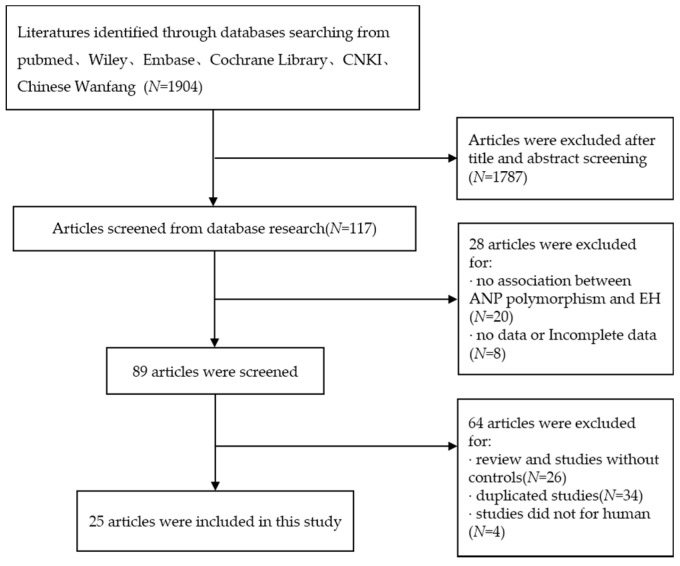
Flow chart of studies included in this meta-analysis.

According to the inclusion and exclusion, a total of 25 studies including 5520 cases and 5210 controls were available for this meta-analysis. The specific flow chart is shown in Figure 1. The basic characteristics of the studies included are presented in Table 1 and Table 2. The HWE test was also conducted to identify the genotype distribution of the controls in all of the studies. Three SNPs were analyzed, including T2238C, G1837A and T1766C, in 25 studies. Among the studies included in this meta-analysis, 15 articles explored the relationship between hypertension and T2238C polymorphism, six articles were about G1837A polymorphism and four articles were about T1766C polymorphism. Stratification occurred according to the source of subjects; two design methods were conducted including (P-B) population-based and (H-B) hospital-based; according to the ethnicity of the subjects, three races were considered, including Asian, White and Black. The four genotyping methods included PCR, polymerase chain reaction and restriction fragment length polymorphism (PCR-RFLP), gene chips and Q-PCR.

### 3.2. Meta-Analysis

**Figure 2:**
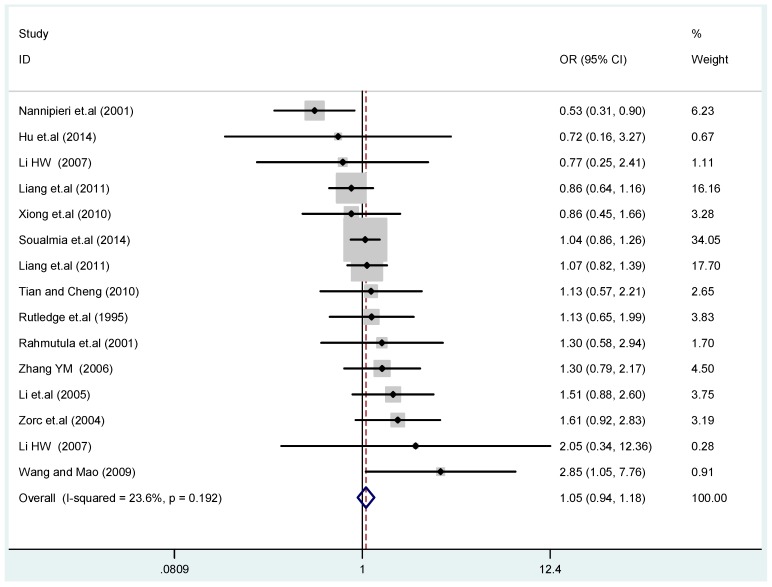
Forest plot of overall population of T2238C additive model (C *vs.* T).

**Figure 3:**
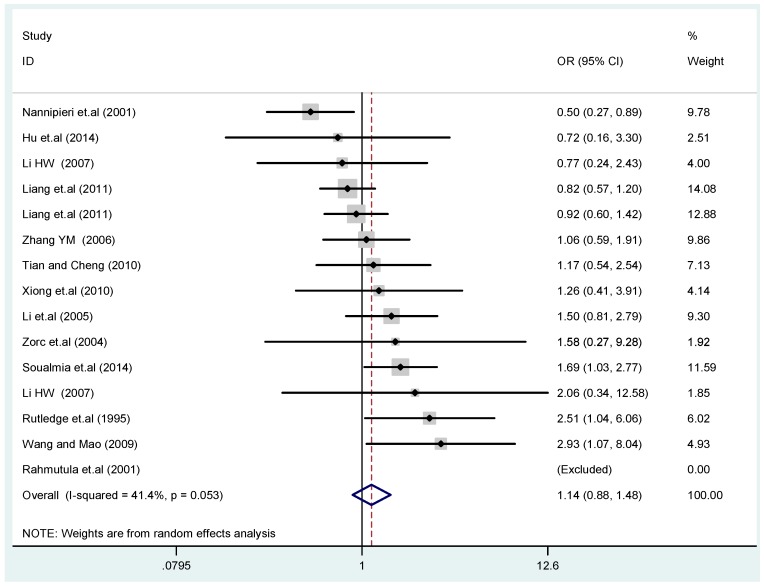
Forest plot of overall population of T2238C co-dominant model-1 (TC *vs.* TT).

**Figure 4:**
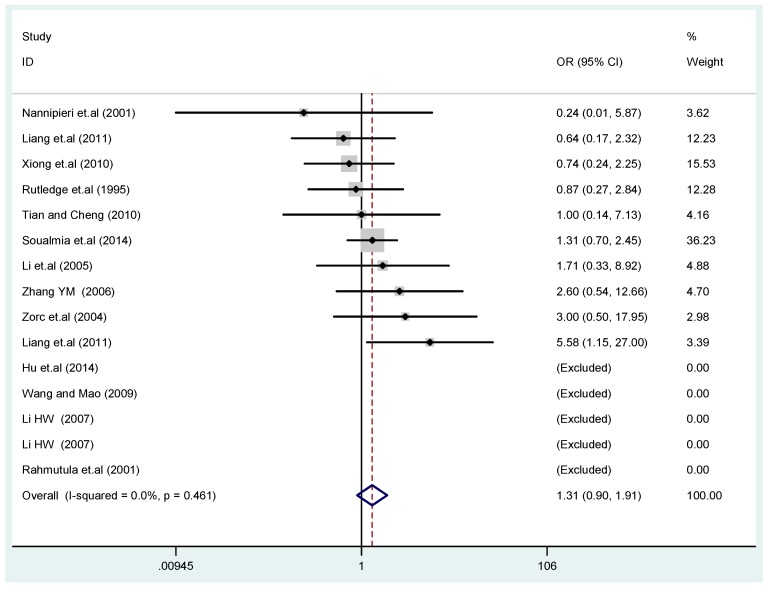
Forest plot of overall population of T2238C co-dominant model-2 (CC *vs.* TT).

**Figure 5:**
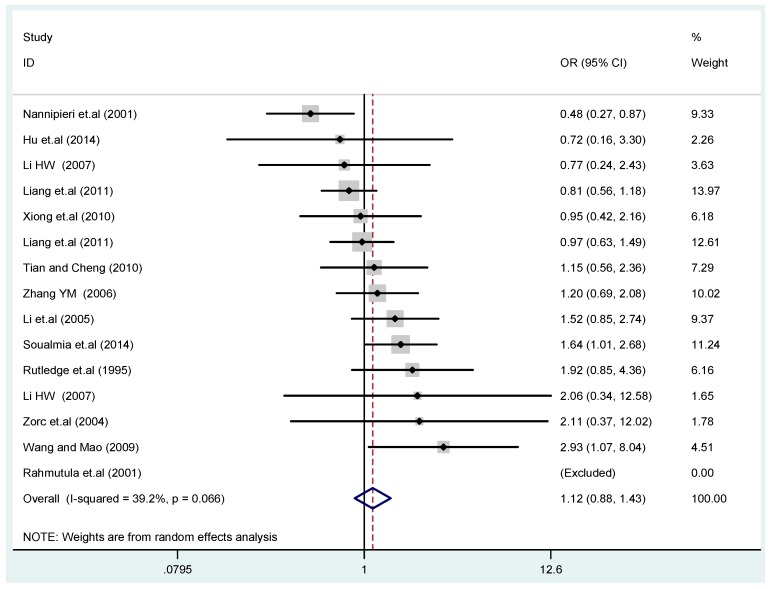
Forest plot of overall population of T2238C dominant model (CC + TC) *vs.* TT.

**Figure 6:**
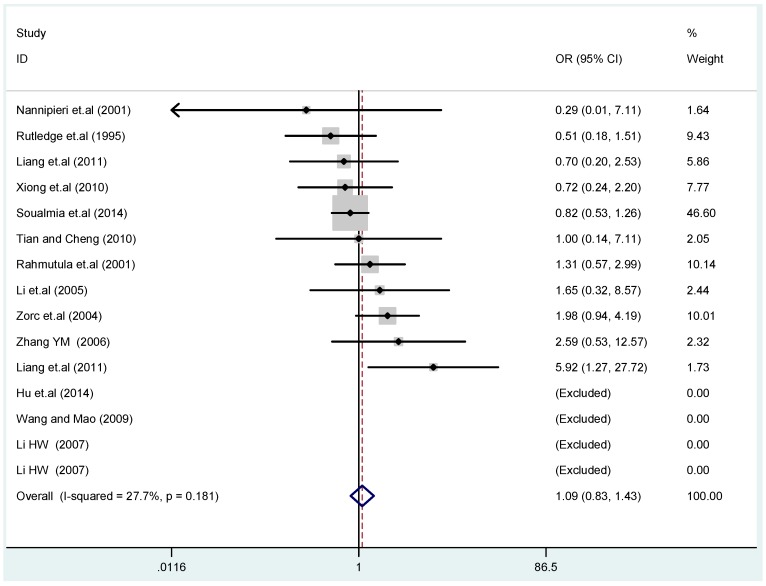
Forest plot of overall population of T2238C recessive model CC *vs.* (TT + TC).

The results of the heterogeneity test of the total population of the association between T2238C polymorphisms and EH were as follows: C *vs.* T: p* = 0.19, *I*^2^ = 23.6%; TC *vs.* TT: p* = 0.053, *I*^2^ = 41.4%; CC *vs.* TT: p* = 0.46, *I*^2^ = 0.0%; (CC + TC) *vs.* TT: p* = 0.066, *I*^2^ = 39.2%; CC *vs.* (TT + TC): p* = 0.18, *I*^2^ = 27.7% (p*: *p* value of heterogeneity). The results of the test for heterogeneity of the overall population of G1837A polymorphisms and EH were as follows: A *vs.* G: p* = 0.051, *I*^2^ = 54.7%; GA *vs.* GG: p* = 0.005, *I*^2^ = 70.1%; AA *vs.* GG: p* = 0.48, *I*^2^ = 0.0%; (AA + GA) *vs.* GG: p* = 0.009, *I*^2^ = 67.5%; AA *vs.* (GG + GA): p* = 0.53, *I*^2^ = 0.0% (p*: *p* value of heterogeneity). In the overall population, if the test level α = 0.10, in the T2238C polymorphism analysis, except for the co-dominant model (TC *vs.* TT) and dominant model ((CC + TC) *vs.* TT), the other three models all met the level *p* > 0.10 and *I*^2^ < 50%; a random effects model was used in the co-dominant model (TC *vs.* TT) and dominant model ((CC + TC) *vs.* TT), and a fixed effects model was conducted in the other three genetic models. The forest plots of five genetic models of the total population between T2238C polymorphism and EH are presented in Figure 2, Figure 3, Figure 4, Figure 5 and Figure 6; the P value of significance test(s) of OR = 1 is shown in Table 3. Overall, no statistically significant associations between T2238C polymorphisms and EH were found in five models of the total population. The results of meta-analysis of the G1837A polymorphism and EH are shown in Table 4; five genetic models of the overall population were also conducted. The results of meta-analysis of the T1766C polymorphism and EH are presented in Table 5.

### 3.3. Sensitivity Analysis

A sensitivity analysis was performed on three gene loci to evaluate the influence of each individual study on the pooled OR. The sensitivity analysis of the T2238C polymorphism showed that none of the fifteen studies included in this meta-analysis dramatically influenced the combined results under all of the five genetic models. The sensitivity analysis of the G1837A polymorphism suggested that Rutledge [30] significantly influenced the combined results under all of the five genetic models; the tests for heterogeneity changed significantly if the study of Rutledge was removed. The results indicated that the source of heterogeneity may be caused by ethnicity, as shown in Table 4 and Table 6.

**Figure 7:**
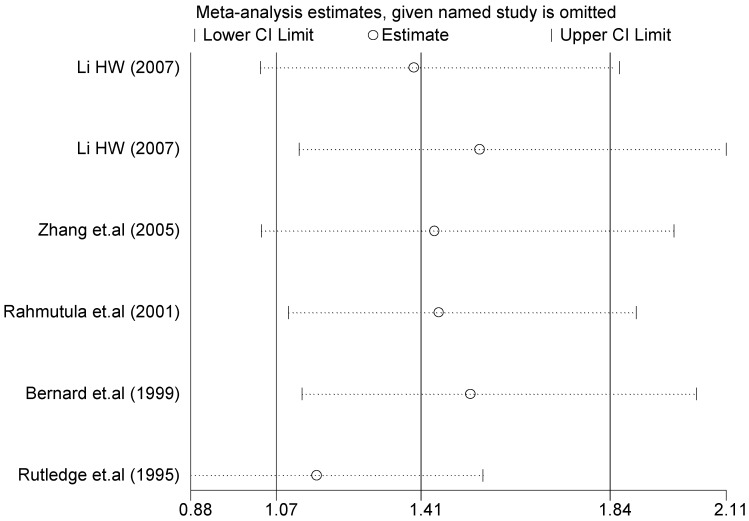
Forest plot of sensitivity analysis of overall population of G1837A polymorphism and EH (GA *vs.* GG).

**Figure 8:**
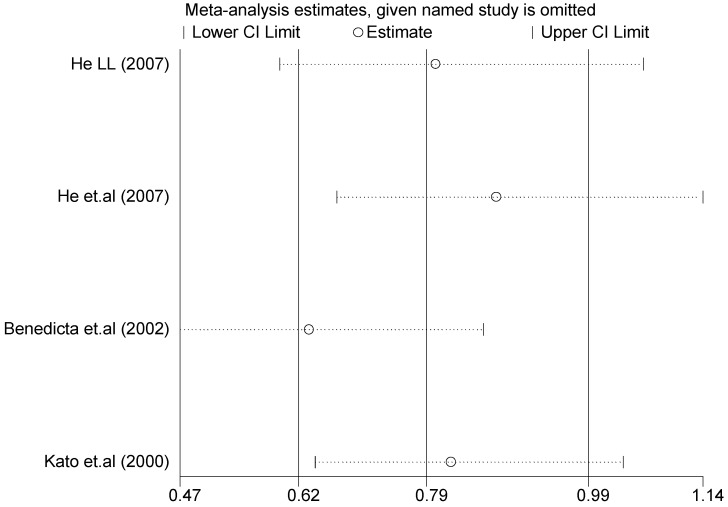
Forest plot of sensitivity analysis of overall population of T1766C polymorphism and EH (TC *vs.* TT).

The forest plot of sensitivity analysis is presented in Figure 7. Because the study of Rutledge [30] met the inclusion criteria, stricter interpretation needs to be conducted. Through the sensitivity analysis of T1766C polymorphism, the results become statistically significant under the genetic models of (TC *vs.* TT) and ((CC + TC) *vs.* TT) after excluding the study of Benedicta [35]. The results are shown in Table 5 and Table 7, and the forest plot of sensitivity analysis is presented in Figure 8. The results of this study need to be interpreted carefully because this study met the inclusion criteria.

### 3.4. Subgroup Analysis

**Figure 9:**
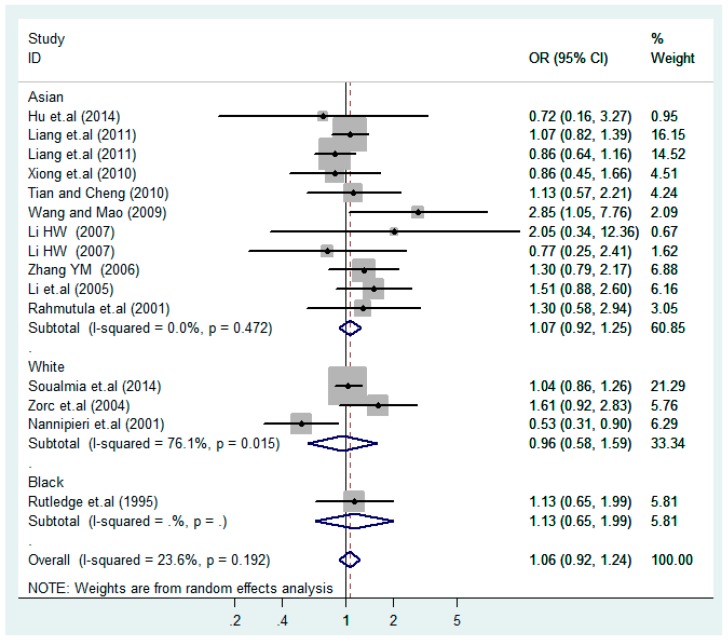
Ethnic subgroups analysis of T2238C additive model (C *vs.* T).

**Figure 10:**
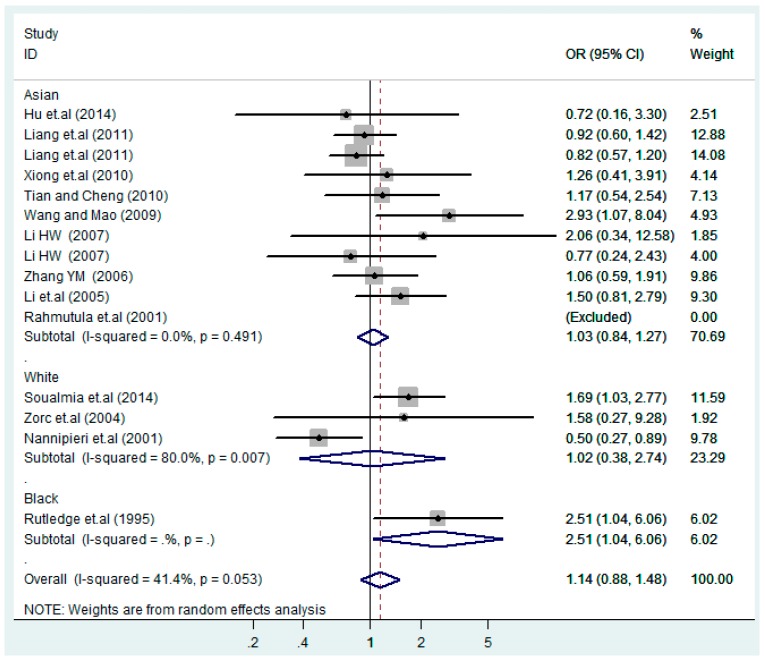
Ethnic subgroups analysis of T2238C co-dominant model-1 (TC *vs.* TT).

**Figure 11:**
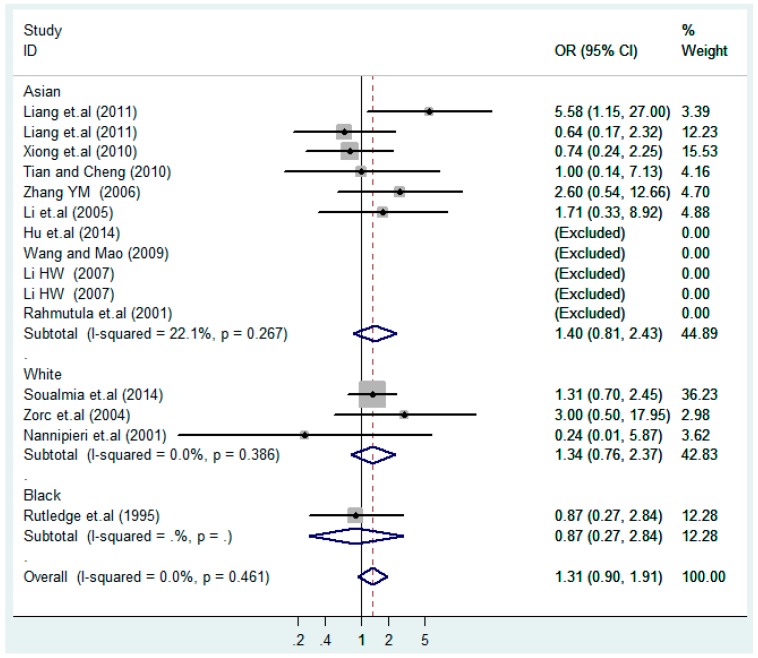
Ethnic subgroups analysis of T2238C co-dominant model-2 (CC *vs.* TT).

**Figure 12:**
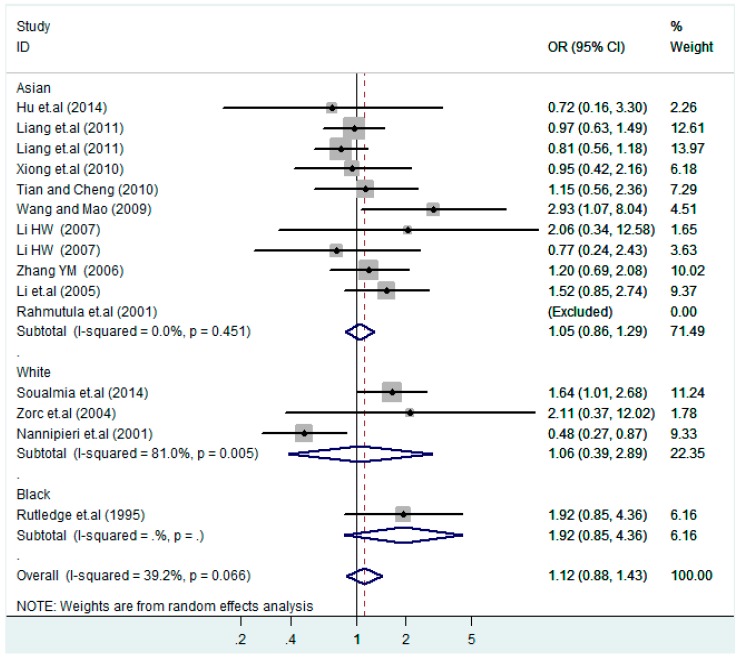
Ethnic subgroup analysis of T2238C dominant model (CC + TC) *vs.* TT.

**Figure 13:**
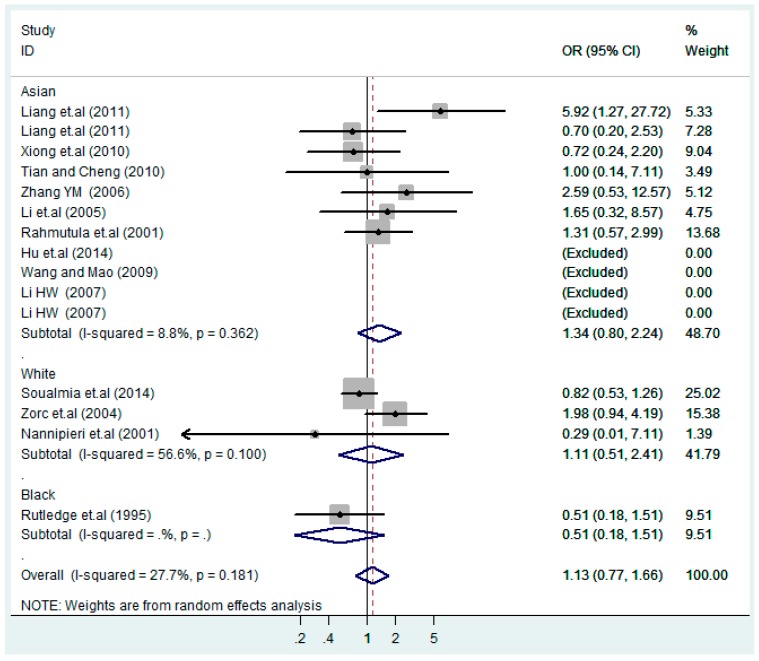
Ethnic subgroup analysis of T2238C recessive model CC *vs.* (TT + TC).

**Figure 14:**
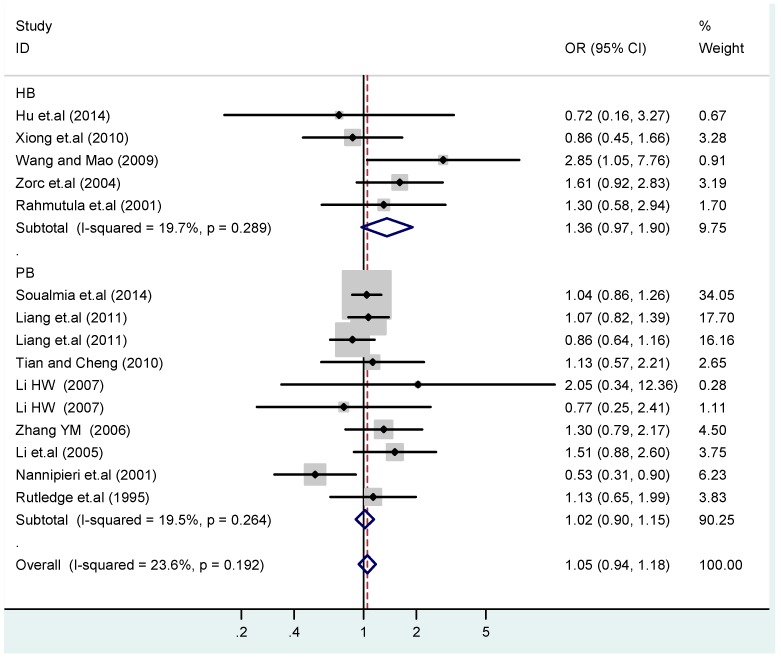
Subgroups in sources of controls of T2238C additive model (C *vs.* T).

**Figure 15:**
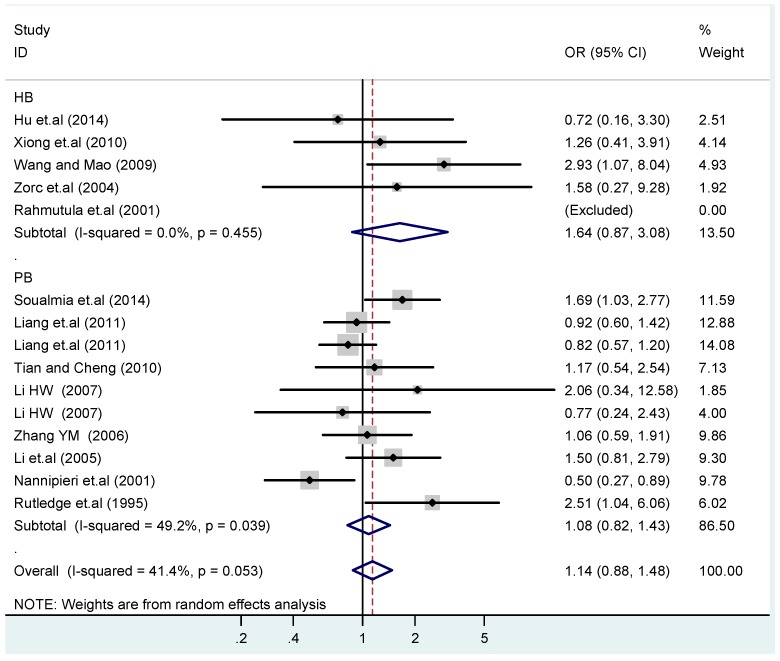
Subgroups in sources of controls of T2238C co-dominant model-1 (TC *vs.* TT).

**Figure 16:**
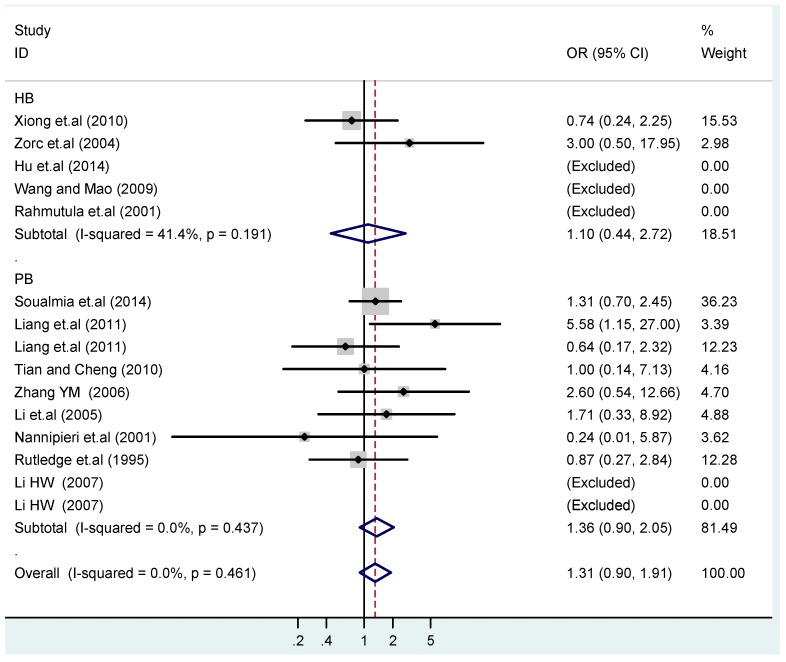
Subgroups in sources of controls of T2238C co-dominant model-2 (CC *vs.* TT).

**Figure 17:**
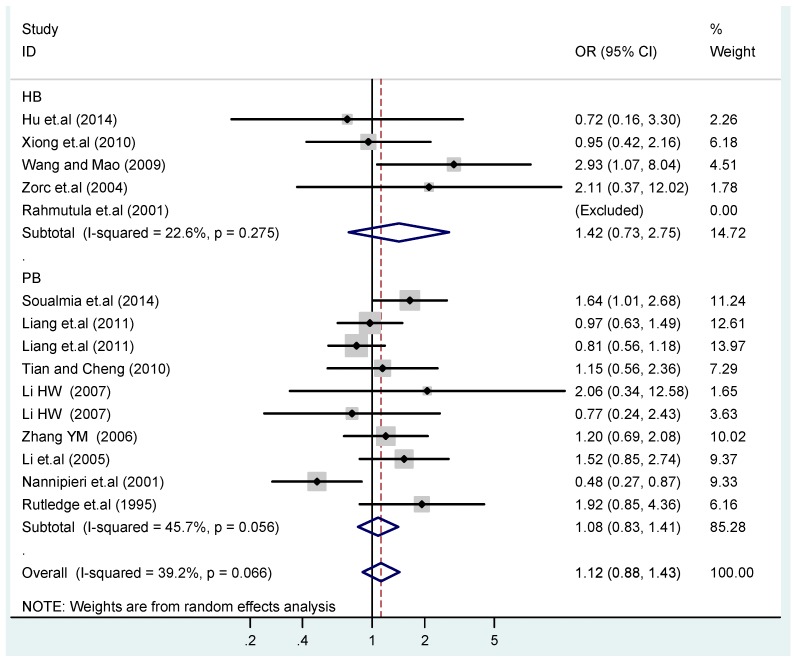
Subgroups in sources of controls of T2238C dominant model (CC + TC) *vs.* TT.

**Figure 18:**
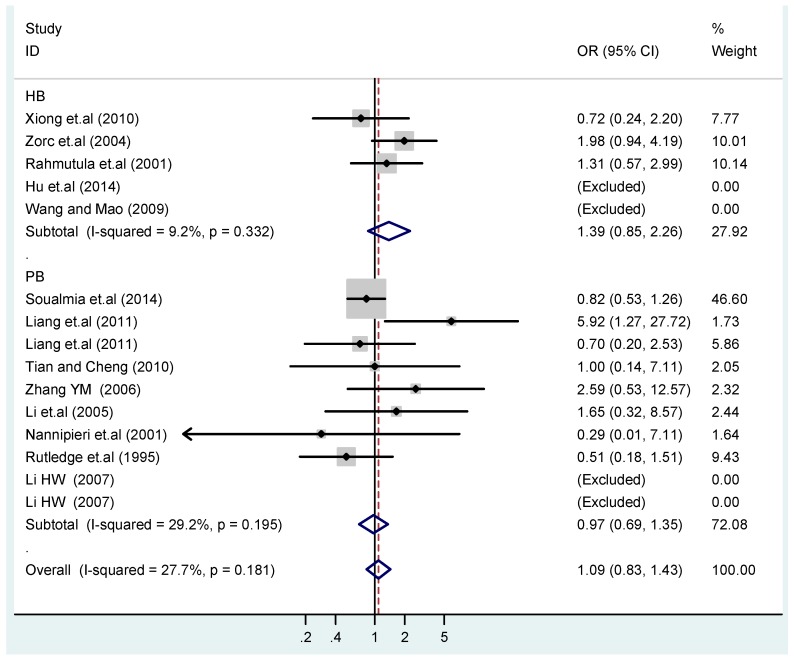
Subgroups in sources of controls of T2238C recessive model CC *vs.* (TT + TC).

**Figure 19:**
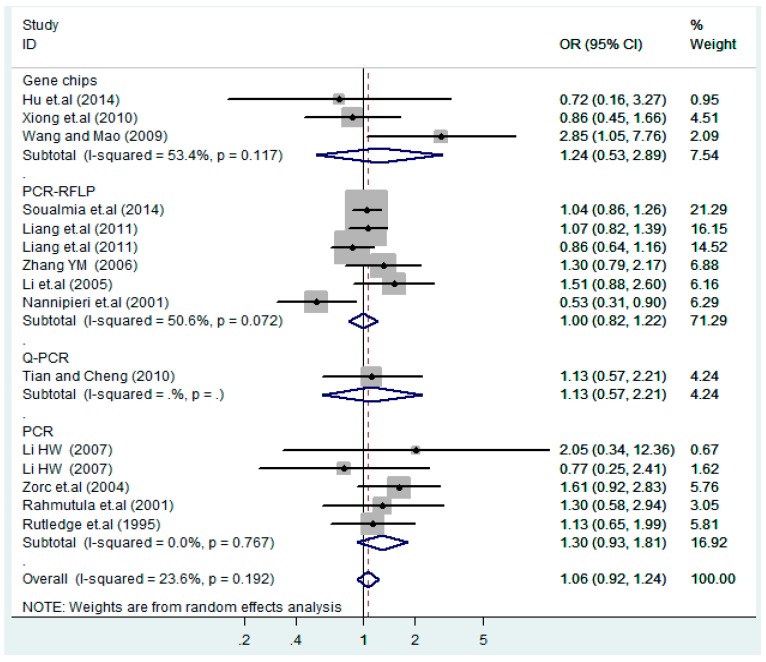
Subgroups of genotyping methods of T2238C additive model (C *vs.* T).

**Figure 20:**
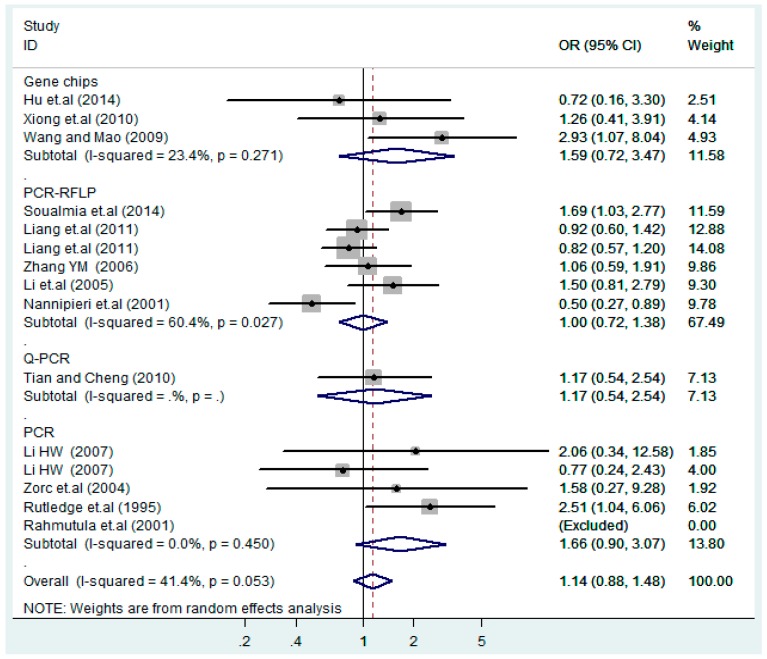
Subgroups of genotyping methods of T2238C co-dominant model-1 (TC *vs.* TT).

**Figure 21:**
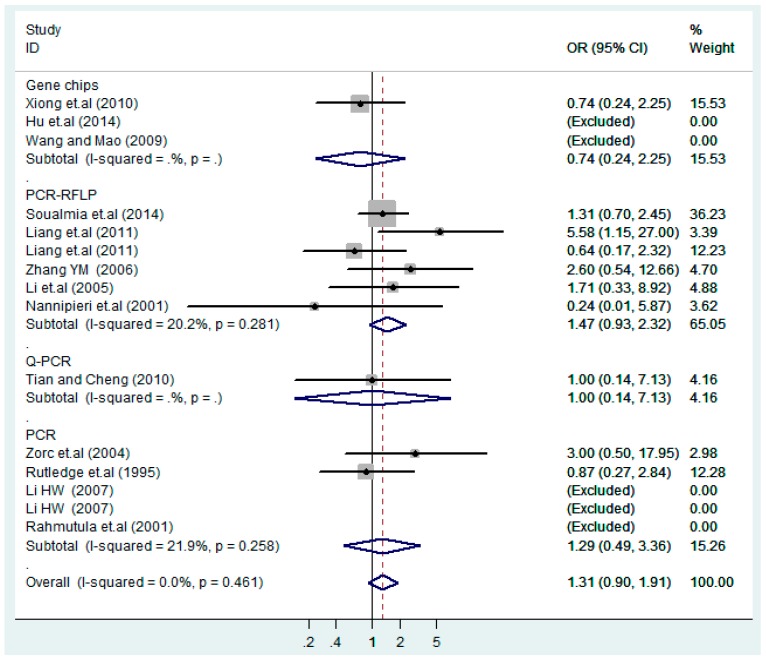
Subgroups of genotyping methods of T2238C co-dominant model-2 (CC *vs.* TT).

**Figure 22:**
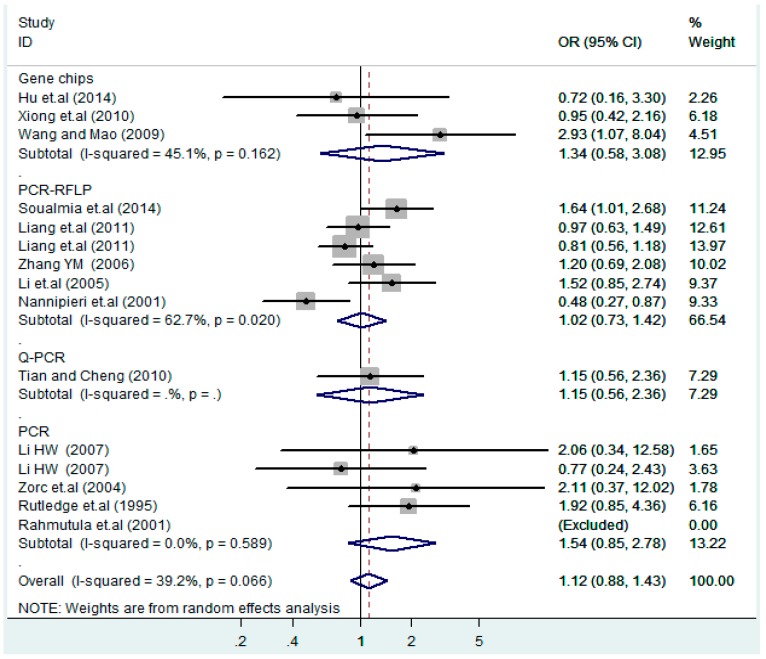
Subgroups of genotyping methods of T2238C dominant model (CC + TC) *vs.* TT.

**Figure 23:**
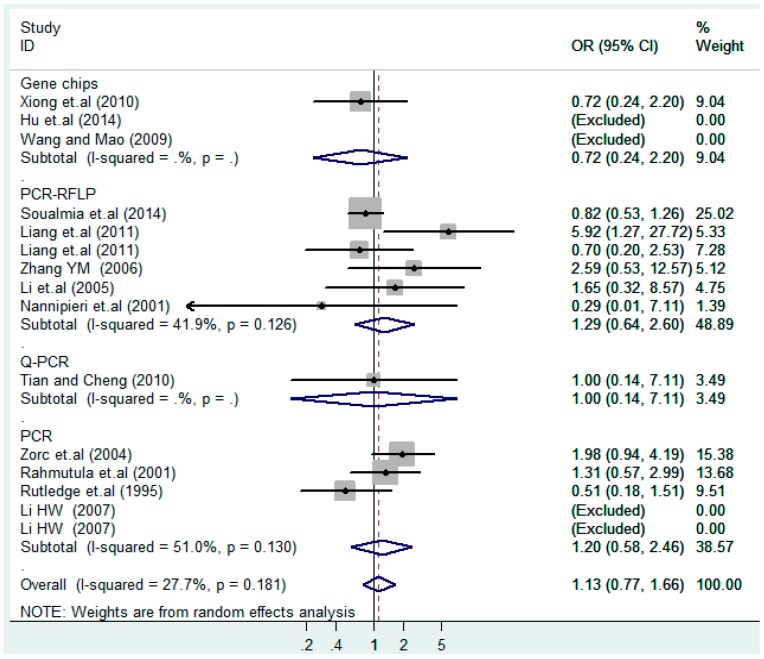
Subgroups of genotyping of T2238C recessive model CC *vs.* (TT + TC).

According to the ethnicity of subjects, genotyping methods, and the source of controls, a stratified analysis was performed on the T2238C polymorphism to explore the sources of heterogeneity as follows. In the test for heterogeneity of ethnic subgroups in the T2238C additive model, the P value of heterogeneity in Asian and White subgroups was 0.472 and 0.015, respectively, and the *I*^2^ in Asian and White subgroups was 0.0% and 76.1%, respectively, so a random effects model was conducted to estimate the summary OR and corresponding 95%CI. Ethnic subgroup analysis of the association between T2238C polymorphisms and EH of the other four models is shown in Figure 9, Figure 10, Figure 11, Figure 12 and Figure 13 and Table 3. According to the test for heterogeneity of the subgroups analysis in the sources of controls of the T2238C additive model (C *vs.* T), the *p* value of heterogeneity in HB and PB was 0.289 and 0.264, respectively, and the *I*^2^ in HB and PB was 19.7% and 19.5%, respectively, so a fixed effects model was conducted to estimate the summary OR and corresponding 95%CI. The subgroup analysis of the sources of controls of the association between T2238C polymorphisms and EH of the other four models are shown in Figure 14, Figure 15, Figure 16, Figure 17 and Figure 18 and Table 3. In the subgroup analysis of genotyping methods of the association between T2238C polymorphisms and EH, the fixed effects model was used in the co-dominant model-2 (CC *vs.* TT), and a random effects model was conducted in the other four models. The forest plots of the five models and the results of the meta-analysis are shown in Figure 19, Figure 20, Figure 21, Figure 22 and Figure 23 and Table 3.

### 3.5. Publication Bias

**Figure 24:**
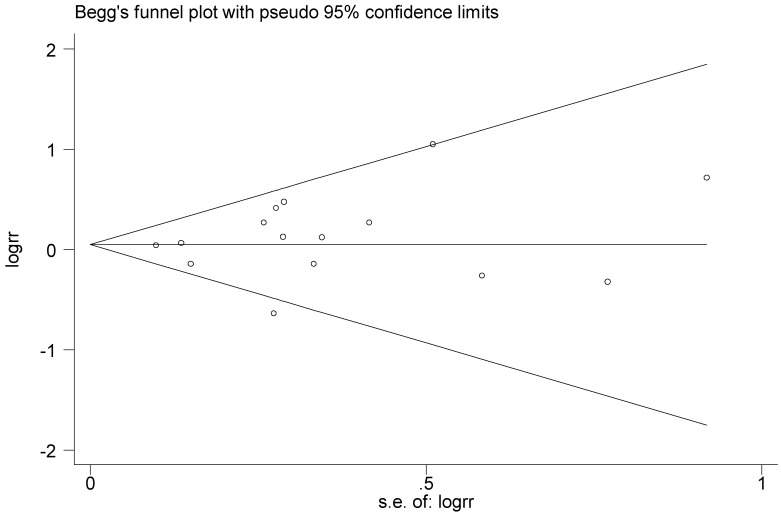
Begg’s funnel plot of T2238C polymorphism for the publication bias test (C *vs.* T).

An evaluation of publication bias of T2238C polymorphism was conducted for the 15 articles included in this meta-analysis. No obvious publication bias was found in the meta-analysis under the five genetic models. Both Begg’s test and Egger’s test were conducted. The *p* values of Begg’s and Egger’s test under the five genetic models all satisfied *p* > 0.1, the results indicated that there was no significant publication bias, and Begg’s test funnel plot was drawn, as seen in Figure 24.

## 4. Discussion

Many gene loci in human ANP gene associated with essential hypertension have been found, including the T2238C, G1837A, T1766C, C664G, C1364A, G658A and G664A gene polymorphisms. Robert explored the relationship between the ANF gene and essential hypertension in terms of causation. However, the results provided no evidence for the involvement of the ANF gene polymorphism with EH [37]. Rutledge investigated gene polymorphisms within the atrial natriuretic peptide of African Americans at intron two and exon three in essential hypertension and found that the *Hpa*II polymorphism was associated with hypertension [30]. Cheung discovered that the allele distribution H1 and H2 of the *Hpa*II polymorphism of the atrial natriuretic peptide gene in hypertensive patients and normotensive controls were 0.12 and 0.88, and 0.11 and 0.89, respectively. The results indicated no obvious association with hypertension in this population [32]. Zorc-Pleskovic analyzed the T2238C *Sca*I gene polymorphism of the ANF gene in a group of children with EAH, and the results also failed to find an association between the T2238C gene polymorphism and EH in children [27]. In our study, no obvious association was found in the gene locus of T2238C, G1837A and EH.

More epidemiological studies investigating the correlation between the atrial natriuretic peptide (ANP) genetic polymorphism and the risk of essential hypertension worldwide have emerged. However, these studies have reported inconsistent, even contradictory results. Considering the limited sample size of individual studies and the great clinical heterogeneity, meta-analysis can provide a more reliable estimation using quantitative synthesis methods. A meta-analysis can collect all the relevant studies published or unpublished systematically and comprehensively. The aim of this meta-analysis is to make a more reliable estimation of the possible relationship between the atrial natriuretic peptide genetic polymorphism and the risk of essential hypertension. A few meta-analyses have been conducted to explore the associations between the ANP gene polymorphisms and the risk of EH. However, this meta-analysis is the first to collect relevant articles published on three common gene loci of the ANP gene polymorphism and EH. Although Niu [38] conducted a meta-analysis of the relationship between a natriuretic peptide precursor, the T2238C polymorphism and hypertension, the articles included were limited and the gene locus only included the T2238C polymorphism. In Niu [38], only seven studies were included; the results indicated that the 2238C allele decreased risk of developing hypertension, a results that is inconsistent with the results of this meta-analysis.

**Figure 25:**
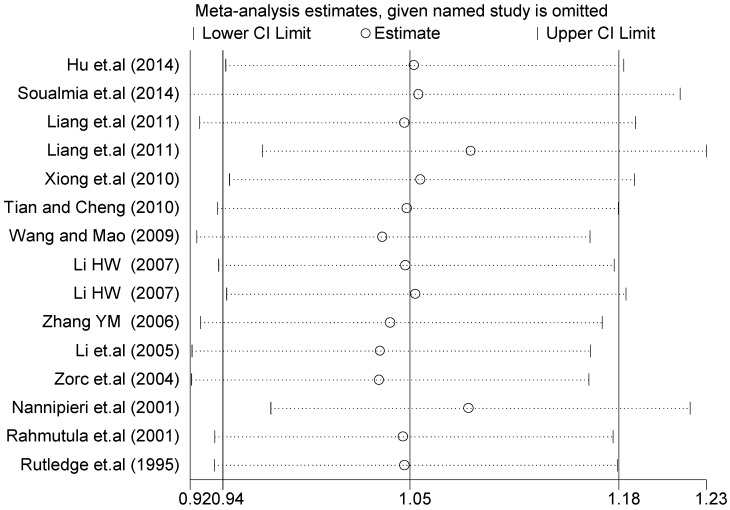
Forest plot of sensitivity analysis of overall population of T2238C polymorphism and EH (C *vs.* T).

A sensitivity analysis was also conducted in the present study on three gene loci to evaluate the influence of each individual study on the pooled OR. The sensitivity analysis of the T2238C polymorphism showed that none of the fifteen studies included in this meta-analysis substantially influenced the combined results under all five genetic models. The forest plot of sensitivity analysis of the overall population of the T2238C polymorphism is shown in Figure 25. The sensitivity analysis of the G1837A polymorphism suggested that Rutledge [30] significantly influenced the combined results under all five genetic models; the tests for heterogeneity changed significantly if the study of Rutledge [30] was removed. The ethnicity of Rutledge [30] was Black, and the remaining five studies were all Asians, which indicated that the source of heterogeneity may be caused by ethnicity. Through sensitivity analysis of the T1766C polymorphism, the results become statistically significant under the genetic models of (TC *vs.* TT) and ((CC + TC) *vs.* TT) after excluding the study of Benedicta [35]. Therefore, the results of this study need to be interpreted carefully.

In the meta-analysis of Niu [38], the subgroup analysis of the T2238C polymorphism by study design presented opposite results for the HB and PB groups. However, in the subgroup analysis of this meta-analysis of the ANP T2238C polymorphism by the ethnicity of subjects, no obvious association was found in Asians, Whites and Blacks under the five genetic models in the overall population. Moreover, in the subgroup analysis of the ANP T2238C polymorphism by genotyping methods, no significant difference was found in PCR, PCR-RFLP, gene chips and Q-PCR under the five genetic models. Similarly, in the subgroup analysis of the ANP T2238C polymorphism by the source of controls, there was no apparent association between the T2238C polymorphism and EH in the (PB) population-based and (HB) hospital-based controls under the five genetic models. The forest plots in the subgroup analysis of the ANP T2238C polymorphism by the ethnicity of subjects, genotyping methods and the source of controls under the five genetic models were presented in Figure 9, Figure 10, Figure 11, Figure 12, Figure 13, Figure 14, Figure 15, Figure 16, Figure 17, Figure 18, Figure 19, Figure 20, Figure 21, Figure 22 and Figure 23.

## 5. Conclusions

In conclusion, this meta-analysis indicates that the ANPT2238C, G1837A gene polymorphism may have no relationship with EH; conversely, the ANP T1766C gene polymorphism is likely to be associated with EH. Considering the limited articles included in this meta-analysis of T1766C polymorphism, more articles are needed for future studies. According to the sensitivity analysis and publication bias evaluation, no obvious publication bias was found, which indicates that the conclusion of this article is basically reliable and stable.

## Figures and Tables

**Table 1:** Characteristics of studies included in the meta-analysis.

Author	Year	Locus	Source		Country	Ethnicity	Number		Genotyping Methods	Quality Score
		T2238C	Case	Control			Case	Control		
Hu *et al.* [17]	2014		H-B	H-B	China	Asian (Han)	100	97	Gene chips	6
Soualmia *et al.* [14]	2014		H-B	P-B	Tunisia	White (Tunisian)	384	453	PCR-RFLP	7
Liang *et al.* [20]	2011		P-B	P-B	China	Asian (Han)	205	260	PCR-RFLP	6
Liang *et al.* [20]	2011		P-B	P-B	China	Asian (Kazakh)	218	232	PCR-RFLP	6
Xiong *et al.* [21]	2010		H-B	H-B	China	Asian (Han)	81	120	Gene chips	5
Tian and Cheng [22]	2010		H-B	P-B	China	Asian (Han)	976	976	Q-PCR	6
Wang and Mao [23]	2009		H-B	H-B	China	Asian (Han)	238	184	Gene chips	4
Li [24]	2007		P-B	P-B	China	Asian (Yi)	99	134	PCR	5
Li [24]	2007		P-B	P-B	China	Asian (Hani)	172	133	PCR	5
Zhang YM [25]	2006		P-B	P-B	China	Asian (Kazakh)	314	229	PCR-RFLP	6
Li *et.al.* [26]	2005		P-B	P-B	China	Asian (Kazakh)	313	205	PCR-RFLP	6
Zorc *et.al.* [27]	2004		H-B	H-B	Slovenia	Caucasian	58	57	PCR	6
Nannipieri *et.al.* [28]	2001		P-B	P-B	Europeans	White	121	105	PCR-RFLP	6
Rahmutula *et.al.* [29]	2001		H-B	H-B	Japan	Asian	233	213	PCR	3
Rutledge *et.al.* [30]	1995		H-B	P-B	American	Black	60	44	PCR	6
		G1837A	case	control			case	control		
Li [24]	2007		P-B	P-B	China	Asian (Yi)	99	134	PCR	5
Li [24]	2007		P-B	P-B	China	Asian (Hani)	172	133	PCR	5
Zhang *et.al.* [31]	2005		P-B	P-B	China	Asian (Kazakh)	287	190	PCR-RFLP	5
Rahmutula *et.al.* [29]	2001		H-B	H-B	Japan	Asian	233	213	PCR	3
Bernard *et.al.* [32]	1999		H-B	P-B	China	Asian	108	109	PCR	6
Rutledge *et.al.* [30]	1995		H-B	P-B	American	Black	60	44	PCR	6
		T1766C	case	control			case	control		
He [33]	2007		P-B	P-B	China	Asian (Kazakh)	199	198	PCR-RFLP	5
He *et.al.* [34]	2007		P-B	P-B	China	Asian (Kazakh)	246	244	PCR-RFLP	5
Benedicta *et.al.* [35]	2002		H-B	P-B	African	Black	289	278	PCR-RFLP	6
Kato *et.al.* [36]	2000		H-B	H-B	Japan	Asian	255	225	PCR	5

P-B: population-based; H-B: hospital-based.

**Table 2:** The allele gene and genotype frequency of ANP polymorphisms in the meta-analysis.

Author	Year	Locus	Allele Number				Gene Number						HWE
			Case		Control		Case			Control			
		T2238C	T	C	T	C	TT	TC	CC	TT	TC	CC	
Hu *et.al.* [17]	2014		197	3	190	4	97	3	0	93	4	0	YES
Soualmia *et.al.* [14]	2014		372	396	448	458	27	318	39	50	348	55	NO
Liang *et.al.* [20]	2011		246	164	320	200	50	146	9	62	196	2	YES
Liang *et.al.* [20]	2011		322	114	329	135	108	106	4	103	123	6	YES
Xiong *et.al.* [21]	2010		146	16	213	27	70	6	5	103	7	10	YES
Tian and Cheng [22]	2010		1934	18	1936	16	960	14	2	962	12	2	YES
Wang and Mao [23]	2009		458	18	363	5	220	18	0	179	5	0	YES
Li [24]	2007		195	3	266	2	96	3	0	132	2	0	YES
Li [24]	2007		338	6	260	6	166	6	0	127	6	0	YES
Zhang YM [25]	2006		584	44	433	25	277	30	7	206	21	2	YES
Li *et.al.* [26]	2005		581	45	390	20	273	35	5	187	16	2	YES
Zorc *et.al.* [27]	2004		30	86	41	73	2	26	30	4	33	20	YES
Nannipieri *et.al.* [28]	2001		216	26	171	39	95	26	0	67	37	1	YES
Rahmutula *et.al.* [29]	2001		11	455	13	413	0	11	222	0	13	200	YES
Rutledge *et.al.* [30]	1995		70	50	54	34	17	36	7	19	16	9	YES
		G1837A	G	A	G	A	GG	GA	AA	GG	GA	AA	
Li [24]	2007		178	20	245	23	79	20	0	113	19	2	YES
Li [24]	2007		296	48	233	33	127	42	3	101	31	1	YES
Zhang *et.al.* [31]	2005		514	60	346	34	228	58	1	158	30	2	YES
Rahmutula *et.al.* [29]	2001		42	424	47	379	3	36	194	1	45	167	YES
Bernard *et.al.* [32]	1999		191	25	195	23	86	19	3	87	21	1	YES
Rutledge *et.al.* [30]	1995		90	30	85	3	30	30	0	41	3	0	YES
		T1766C	T	C	T	C	TT	TC	CC	TT	TC	CC	
He [33]	2007		304	94	291	105	108	88	3	95	101	2	YES
He *et.al.* [34]	2007		290	202	267	221	49	192	5	29	209	6	YES
Benedicta *et.al.* [35]	2002		333	245	311	245	87	159	43	85	141	52	YES
Kato *et.al.* [36]	2000		506	4	440	10	251	4	0	215	10	0	YES

**Table 3:** Meta-analysis of T2238C polymorphism and EH.

Stratification Factors	No.	Additive Model (C *vs.* T)	*p*	Co-Dominant Model-1 (TC *vs.* TT)	*p*	Co-Dominant Model-2 (CC *vs.* TT)	*p*	Dominant Model (CC + TC) *vs.* TT	*p*	Recessive Model CC *vs.* (TT + TC)	*p*
		OR(95%CI) ^a^		OR(95%CI) ^a^		OR(95%CI) ^a^		OR(95%CI) ^a^		OR(95%CI) ^a^	
Overall	15	1.1(0.94–1.2)	0.38	1.1(0.88–1.5)	0.32	1.3(0.90–1.9)	0.16	1.1(0.88–1.4)	0.35	1.1(0.83–1.4)	0.55
Ethnicity											
Asian	11	1.1(0.92–1.3)	0.38	1.0(0.84–1.3)	0.75	1.4(0.81–2.4)	0.23	1.1(0.86–1.3)	0.62	1.3(0.81–2.2)	0.26
White	3	0.96(0.58–1.6)	0.89	1.0(0.38–2.7)	0.96	1.3 (0.76–2.4)	0.32	1.1(0.39–2.9)	0.91	1.1(0.51–2.4)	0.80
Black	1	1.1(0.65–2.0)	0.66	2.5(1.0–6.1)	0.040	0.87(0.27–2.8)	0.82	1.9(0.85–4.4)	0.12	0.51(0.18–1.5)	0.23
Source of controls											
HB	5	1.4(0.97–1.9)	0.073	1.6(0.87–3.1)	0.13	1.1(0.45–2.7)	0.83	1.4(0.73–2.8)	0.30	1.4(0.85–2.3)	0.19
PB	10	1.0(0.90–1.1)	0.77	1.1(0.82–1.4)	0.58	1.4(0.90–2.1)	0.15	1.1(0.83–1.4)	0.57	0.97(0.70–1.4)	0.86
Genotyping methods											
Gene chips	3	1.2(0.53–2.9)	0.62	1.6(0.72–3.5)	0.25	0.74(0.24–2.2)	0.59	1.3(0.58–3.1)	0.50	0.72(0.24–2.2)	0.57
PCR-RFLP	6	1.0(0.82–1.2)	1.0	1.0(0.72–1.4)	0.99	1.5(0.93–2.3)	0.096	1.0(0.73–1.4)	0.91	1.3(0.64–2.6)	0.48
Q-PCR	1	1.1(0.57–2.2)	0.73	1.2(0.54–2.5)	0.69	1.0(0.14–7.1)	1.0	1.1(0.56–2.4)	0.71	1.0(0.14–7.1)	1.0
PCR	5	1.3(0.93–1.8)	0.13	1.7(0.90–3.1)	0.10	1.3(0.49–3.4)	0.61	1.5(0.85–2.8)	0.15	1.2(0.58–2.5)	0.62

**^a^**: pooled OR and relevant 95%CI; *p*: *p* value of significance test(s) of OR = 1.

**Table 4:** Meta-analysis of G1837A polymorphism and EH.

Gene Type	Genetic Model	OR ^a^	95%CI	*p*	*I*^2^	p*	Model
Overall							
A *vs.* G	additive model	1.3	0.96–1.9	0.090	54.7%	0.051	RE
GA *vs.* GG	co-dominant model-1	1.5	0.83–2.6	0.19	70.1%	0.005	RE
AA *vs.* GG	co-dominant model-2	0.87	0.34–2.3	0.78	0.0%	0.48	FE
(AA + GA) *vs.* GG	dominant model	1.5	0.86–2.5	0.17	67.5%	0.009	RE
AA *vs.* (GG + GA)	recessive model	1.3	0.85–2.0	0.22	0.0%	0.53	FE
Work of Rutledge removed							
A *vs.* G	additive model	1.2	0.95–1.5	0.14	0.0%	1.0	FE
GA *vs.* GG	co-dominant model-1	1.2	0.88–1.6	0.29	0.0%	0.56	FE
AA *vs.* GG	co-dominant model-2	0.87	0.34–2.3	0.78	0.0%	0.48	FE
(AA + GA) *vs.* GG	dominant model	1.2	0.88–1.5	0.28	0.0%	0.82	FE
AA *vs.* (GG + GA)	recessive model	1.3	0.85–2.0	0.22	0.0%	0.53	FE

**^a^**: pooled OR and relevant 95%CI; *p*: *p* value of significance test(s) of OR = 1; p*: *p* value of heterogeneity; FE: fixed effect model; RE: random effect model.

**Table 5:** Meta-analysis of T1766C polymorphism and EH.

Gene Type	Genetic Model	OR ^a^	95%CI	*p*	*I*^2^	p*	Model
Overall							
C *vs.* T	additive model	0.87	0.75–1.0	0.063	0.0%	0.42	FE
TC *vs.* TT	co-dominant model-1	0.73	0.49–1.1	0.12	58.0%	0.068	RE
CC *vs.* TT	co-dominant model-2	0.78	0.50–1.2	0.29	0.0%	0.66	FE
(CC + TC) *vs.* TT	dominant model	0.73	0.51–1.0	0.084	51.1%	0.11	RE
CC *vs.* (TT + TC)	recessive model	0.79	0.53–1.2	0.26	0.0%	0.77	FE
Work of Benedicta removed							
C *vs.* T	additive model	0.82	0.68–1.0	0.052	8.4%	0.34	FE
TC *vs.* TT	co-dominant model-1	0.64	0.47–0.86	0.003	12.7%	0.32	FE
CC *vs.* TT	co-dominant model-2	0.69	0.25–1.9	0.48	0.0%	0.38	FE
(CC + TC) *vs.* TT	dominant model	0.64	0.48–0.87	0.004	18.1%	0.30	FE
CC *vs.* (TT + TC)	recessive model	0.99	0.37–2.7	0.99	0.0%	0.59	FE

**^a^**: pooled OR and relevant 95%CI; *p*: *p* value of significance test(s) of OR = 1; p*: *p* value of heterogeneity; FE: fixed effect model; RE: random effect model.

**Table 6:** Sensitivity analysis of G1837A polymorphism and EH (GA *vs.* GG).

Study Omitted	Estimate	95%Confidence Interval
Li (2007) [24]	1.4	1.0–1.9
Li (2007) [24]	1.5	1.1–2.1
Zhang *et.al.* (2005) [31]	1.4	1.0–2.0
Rahmutula *et.al.* (2001) [29]	1.4	1.1–1.9
Bernard *et.al.* (1999) [32]	1.5	1.1–2.0
Rutledge *et.al.* (1995) [30]	1.2	0.88–1.6
Combined	1.4	1.1–1.8

**Table 7:** Sensitivity analysis of T1766C polymorphism and EH (TC *vs.* TT).

Study Omitted	Estimate	95%Confidence Interval
He (2007) [33]	0.80	0.60–1.1
He *et.al.*(2007) [34]	0.88	0.67–1.1
Benedicta *et.al.*(2002) [35]	0.64	0.47–0.86
Kato *et.al.*(2000) [36]	0.82	0.65–1.0
Combined	0.79	0.62–0.99

## References

[1] Franceschini N., Reiner A.P., Heiss G. (2011). Recent findings in the genetics of blood pressure and hypertension traits. Am. J. Hypertens.

[2] Kearney P.M., Whelton M., Reynolds K., Muntner P., Whelton P.K., He J. (2005). Global burden of hypertension: Analysis of worldwide data. Lancet.

[3] Hajjar I., Kotchen T.A. (2003). Trends in prevalence, awareness, treatment, and control of hypertension in the United States, 1988–2000. JAMA.

[4] Lloyd-Jones D., Adams R., Carnethon M., De Simone G., Ferguson T.B., Flegal K., Ford E., Furie K., Go A., Greenlund K. (2009). Heart disease and stroke statistics—2009 update: A report from the American Heart Association Statistics Committee and Stroke Statistics Subcommittee. Circulation.

[5] Takahashi N., Smithies O. (1999). Gene targeting approaches to analyzing hypertension. J. Am. Soc. Nephrol..

[6] Ilhan N., Kucuksu M., Kaman D., Ilhan N., Ozbay Y. (2008). The 677 C/T MTHFR polymorphism is associated with essential hypertension, coronary artery disease, and higher homocysteine levels. Arch. Med. Res..

[7] Bulut D., Potthast R., Hanefeld C., Schulz T., Kuhn M., Mügge A. (2003). Impaired vasodilator responses to atrial natriuretic peptide in essential hypertension. Eur. J. Clin. Invest..

[8] Tanira M.O., Al B.K. (2005). Genetic variations related to hypertension: A review. J. Hum. Hypertens.

[9] Nakayama T. (2005). The genetic contribution of the natriuretic peptide system to cardiovascular diseases. Endocr J..

[10] Kishimoto I., Rossi K., Garbers D.L. (2001). A genetic model provides evidence that the receptor for atrial natriuretic peptide (guanylyl cyclase-A) inhibits cardiac ventricular myocyte hypertrophy. Proc. Natl. Acad. Sci. USA.

[11] Rubattu S., De Giusti M., Farcomeni A., Abbolito S., Comito F., Cangianiello S., Greco E.S., Dito E., Pagliaro B., Cotugno M. (2014). T2238C ANP gene variant and risk of recurrent acute coronary syndromes in an Italian cohort of ischemic heart disease patients. J. Cardiovasc. Med. (Hagerstown).

[12] Zhang L., Cheng L., He M., Hu B., Wu T. (2006). ANP T2238C, C-664G gene polymorphism and coronary heart disease in Chinese population. J. Huazhong Univ. Sci. Technolog. Med. Sci..

[13] Qureshi S.F., Ali A., Venkateshwari A., Rao H., Jayakrishnan M.P., Narasimhan C., Shenthar J., Thangaraj K., Nallari P. (2014). Atrial natriuretic peptide gene—A potential biomarker for long QT syndrome. EXCLI J..

[14] Soualmiaa H., Ayadi I., Kallel A. (2014). *Sca*I atrial natriuretic peptide gene polymorphism and hypertension in the Tunisian population. Int. J. Sci. Basic Appl. Res. (IJSBAR).

[15] Conen D., Cheng S., Steiner L.L., Buring J.E., Ridker P.M., Zee R.Y. (2009). Association of 77 polymorphisms in 52 candidate genes with blood pressure progression and incident hypertension: the Women’s Genome Health Study. J. Hypertens..

[16] Zhang S., Mao G., Zhang Y., Tang G., Wen Y., Hong X., Jiang S., Yu Y., Xu X. (2005). Association between human atrial natriuretic peptide Val7Met polymorphism and baseline blood pressure, plasma trough irbesartan concentrations, and the antihypertensive efficacy of irbesartan in rural Chinese patients with essential hypertension. Clin. Ther..

[17] Hu D.C., Zhao X.L., Shao J.C., Wang W., Qian J., Chen A.H., Zhang H.Q., Guo H., Jiang J., Li H.Y. (2014). Interaction of six candidate genes in essential hypertension. Genet Mol. Res..

[18] Rubattu S., Sciarretta S., Volpe M. (2014). Atrial natriuretic peptide gene variants and circulating levels: Implications in cardiovascular diseases. Clin. Sci. (Lond.).

[19] Stang A. (2010). Critical evaluation of the Newcastle-Ottawa scale for the assessment of the quality of nonrandomized studies in meta-analyses. Eur. J. Epidemiol..

[20] Liang X., Xu X., He L. (2011). Association of gene polymorphism of atrial natriuretic peptide with essential hypertension in Han and Kazakh population in Xingjiang. J. Chin. Pract. Diagn. Ther..

[21] Xiong W., Luo Y., Yang C. (2010). Clinical analysis of essential hypertension-related gene polymorphism. Chin. J. Gerontol..

[22] Tian C., Cheng L. (2010). Association of Polymorphisms in Atrial and Brain Natriuretic Peptide Gene with Essential Hypertension. Huazhong Univ. Sci. Technol. (Med. Sci.).

[23] Wang Z., Mao Y. (2009). The relationship between endothelial atrial natriuretic peptide T2238C and type-C natriuretic peptide receptor A-55Cgene polymorphisms and essential hypertension in elderly. J. Clin. Med..

[24] Li H. (2007). The Correlation between Atrial Natriuretic Peptide Gene Single Nucleotide Polymorphism and Essential Hypertention of Hani and Yi Minority Group in Yunnan Province. Ph.D. Thesis.

[25] Zhang Y. (2006). The Relationship Study bewteen ANP Gene and Essential Hypertension in Xinjiang Kazakhs. Ph.D. Thesis.

[26] Li N., Zhang Y., Li T., Zhou L. (2005). The relationship between ANP gene T2238C polymorphism and essential hypertention in xinjiang Kazakans. J. Clin. Cardiol..

[27] Zorc-Pleskovic R., Bidovec M., Bregar D., Milutinović A., Terzić R., Teran N. (2004). The *Sca*I gene polymorphism of the atrial natriuretic factor and essential arterial hypertension in childhood. Coll Antropol..

[28] Nannipieri M., Manganiello M., Pezzatini A., De Bellis A., Seghieri G., Ferrannini E. (2001). Polymorphisms in the hANP (human atrial natriuretic peptide) gene, albuminuria, and hypertension. Hypertension.

[29] Rahmutula D., Nakayama T., Soma M., Takahashi Y., Kunimoto M., Uwabo J., Sato M., Izumi Y., Kanmatsuse K., Ozawa Y. (2001). Association study between the variants of the human ANP gene and essential hypertension. Hypertens Res..

[30] Rutledge D.R., Sun Y., Ross E.A. (1995). Polymorphisms within the atrial natriuretic peptide gene in essential hypertension. J. Hypertens.

[31] Zhang Y., Li N., Li T., Zhou L., Zhang D., Li H., Zhou K. (2005). The relationship bewteen the ANP gene G1837A polymorphism and essential hypertension in Xinjiang Kazakans. Sci. Technol. Engineer.

[32] Cheung B.M., Leung R., Shiu S., Tan K.C.B., Lau C.-P., Kumana C.R. (1999). *Hpa*II polymorphism in the atrial natriuretic peptide gene and hypertension. Am. J. Hypertens.

[33] He L. (2007). Relationship Study bewteen ANP Gene Polymorphism and Essential Hypertension in Xinjiang Kazakhs. Ph.D. Thesis.

[34] He L., Xu X., Liang X. (2007). Association of the T1766C polymorphism of atrial natriuretic peptide gene with essential hypertension in Xinjiang Kazakh. J. Xinjiang Med. Univ..

[35] Nkeh B., Tiago A., Candy G.P., Woodiwiss A.J., Badenhorst D., Luker F., Netjhardt M., Brooksbank R., Libhaber C., Sareli P. (2002). Association between an atrial natriuretic peptide gene polymorphism and normal blood pressure in subjects of African ancestry. Cardiovasc. J. South Afr..

[36] Kato N., Sugiyama T., Morita H., Nabika T., Kurihara H., Yamori Y., Yazaki Y. (2000). Genetic analysis of the atrial natriuretic peptide gene in essential hypertension. Clin. Sci..

[37] Robert Y.L., Lou Z.Y., Griffiths L.R., Griffiths L.R., Morris B.J. (1993). Molecular genetic analyses of RFLPs for PCR-Amplified growth hormone gene, renal kallikrein gene and atrial natriuretic factor gene in essential hypertension. Hypertens Res..

[38] Niu W. (2011). The relationship between natriuretic peptide precursor a gene T2238C polymorphism and hypertension: A Meta-Analysis. Int. J. Hypertens.

